# Lateral wedge insole for knee osteoarthritis: randomized clinical trial: Palmilha valgizante para osteoartrite de joelhos: ensaio clínico randomizado

**DOI:** 10.1590/1516-3180.2013.6750002

**Published:** 2014-10-17

**Authors:** Gustavo Constantino de Campos, Marcia Uchôa Rezende, Thiago Pasqualin, Renato Frucchi, Raul Bolliger

**Affiliations:** I MD. Doctoral Student, Faculdade de Medicina da Universidade de São Paulo (FMUSP), São Paulo, Brazil.; II MD, MSc, PhD. Head of Osteometabolic Disease Group, Hospital das Clínicas (HC), Faculdade de Medicina da Universidade de São Paulo (FMUSP), São Paulo, Brazil.; III MD. Medical Volunteer, Osteometabolic Disease Group, Hospital das Clínicas (HC), Faculdade de Medicina da Universidade de São Paulo (FMUSP), São Paulo, Brazil.; IV MD, MSc, PhD. Attending Physician in Institute of Orthopedics and Traumatology, Hospital das Clínicas (HC), Faculdade de Medicina da Universidade de São Paulo (FMUSP), São Paulo, Brazil.

**Keywords:** Osteoarthritis, Orthotic devices, Randomized controlled trial [publication type], Orthopedics, Knee joint

## Abstract

**CONTEXT AND OBJECTIVE:**

Optimal management of knee osteoarthritis requires a combination of pharmacological and non-pharmacological methods. The use of lateral wedge insoles to treat medial knee osteoarthritis is recommended, but there is still controversy about its efficacy. The purpose of this study was to ascertain whether the use of lateral wedge insoles can diminish pain and improve function in patients with medial knee osteoarthritis.

**DESIGN AND SETTING:**

Prospective randomized trial conducted in a tertiary-level hospital.

**METHODS:**

We prospectively enrolled 58 patients with medial knee osteoarthritis and randomized them to use either a lateral wedge insole with subtalar strapping (Group W), or a neutral insole with subtalar strapping (Group N - control). All the patients were instructed to use the insole for five to ten hours per day. A visual analogue pain scale, the Western Ontario and McMaster Universities Arthritis Index (WOMAC) and the Lequesne questionnaire were applied at baseline and at weeks 2, 8 and 24.

**RESULTS:**

At weeks 8 and 24, both groups showed lower scores for WOMAC (P = 0,023 and P = 0,012 respectively). There were no statistically significant differences between the groups regarding the visual analogue pain scale, WOMAC or Lequesne results at any time evaluated.

**CONCLUSION:**

The use of a lateral wedge insole with subtalar strapping improved the patients’ symptoms and function but was not superior to placebo insoles.

## RESUMO

**CONTEXTO E OBJETIVO:**

O manejo ideal da osteoartrite de joelhos requer combinação entre modalidades farmacológicas e não farmacológicas. O uso de palmilhas valgizantes no tratamento da osteoartrite medial do joelho é recomendado, mas sua eficácia ainda é controversa. Este estudo objetiva verificar se o uso da palmilha valgizante pode diminuir a dor e melhorar a função dos pacientes com osteoartrite medial dos joelhos.

**DESENHO E LOCAL:**

Ensaio clínico prospectivo e randomizado conduzido em hospital de atenção terciária.

**MÉTODOS:**

Alocamos prospectivamente 58 pacientes com osteoartrite medial dos joelhos que foram randomizados para fazer uso de palmilha valgizante com amarrilho subtalar (Grupo W) ou palmilha neutra com amarrilho subtalar (Grupo N - controle). Todos os pacientes foram orientados a utilizar a palmilha entre cinco e dez horas por dia. Foram aplicados os questionários Western Ontario and McMaster Universities Arthritis Index (WOMAC) e Lequesne, além da escala visual analógica da dor, nos momentos pré e após 2, 8 e 24 semanas.

**RESULTADOS:**

Após 8 e 24 semanas, ambos os grupos apresentaram redução dos valores de WOMAC (P = 0,023 e P = 0,012 respectivamente). Não houve diferença estatisticamente significativa entre os grupos nos resultados de WOMAC, Lequesne e escala visual analógica de dor, em nenhum dos momentos avaliados.

**CONCLUSÃO:**

O uso de palmilha valgizante com amarrilho subtalar melhorou os sintomas e a função dos pacientes, mas não foi melhor que placebo.

## INTRODUCTION

Osteoarthritis is a major cause of chronic musculoskeletal pain and disability in the elderly population.^1^ Knee osteoarthritis is one of the most common forms of osteoarthritis and the most frequent chronic condition that leads to functional limitation in older adults, affecting more people than any other joint disease.^2^ Recent guidelines recommend that optimal management of osteoarthritis requires a combination of non-pharmacological and pharmacological methods.^3^ All patients should be given access to information and education about the objectives of treatment and the importance of changes in lifestyle, exercise, pacing of activities, weight reduction and other measures to reduce the load on the damaged joint(s), such as walking aids, knee braces and insoles, as well as muscle strengthening and weight loss.^3^

Involvement of the medial compartment of the knee is ten times more common than involvement of the lateral compartment.^4^^,^^5^ Knee osteoarthritis in the medial compartment is strongly associated with biomechanical factors, particularly progressive varus deformity, which systematically increases the load on the medial compartment, thus further increasing the risk of damage to this compartment.^6^^,^^7^

Biomechanical and clinical studies have shown that lateral wedge insoles can promote a reduction in the adduction moment of 4 to 12% during gait, thus reducing the load on the medial knee compartment^8^^,^^9^^,^^10^ and promoting symptomatic benefit for some patients with medial compartment tibiofemoral osteoarthritis.^11^ The use of lateral wedged insoles for patients with medial compartment knee osteoarthritis is certainly a very interesting treatment option because of its low cost, low complexity and virtually absence of side effects.^12^^,^^13^ However, apart from Japan, where several studies have demonstrated its efficacy,^10^^,^^14^^,^^15^^,^^16^ the recent literature on the use of lateral wedges for medial compartment knee osteoarthritis is insufficient to draw any substantial conclusions. Two recent systematic reviews have shown limited evidence to support the use of lateral wedge orthotics for reducing pain, increasing function or slowing disease progression.^17^^,^^18^

## OBJECTIVE

The purpose of this study was to ascertain whether the use of lateral wedge insoles can diminish pain and improve function in patients with medial knee osteoarthritis.

## METHODS

**Figure 1.**
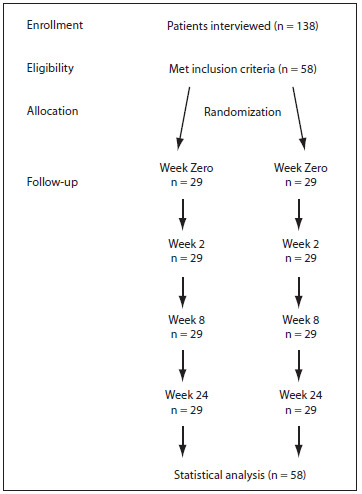
Study flow diagram.

This prospective single-blind parallel group controlled trial was conducted under the principles of the Helsinki Declaration and was approved by CAPPesq (Ethics Committee for analysis of research projects) under the protocol number 839/2011. It followed the CONSORT (Consolidated Standards of Reporting Trials) Statement and evaluated 58 patients with knee osteoarthritis (Figure 1). It was registered at clinicaltrials.gov under the number NCT01739296.

### Eligibility criteria

The eligibility criteria were that patients needed to meet the American College of Rheumatology criteria for knee osteoarthritis^19^ and to present varus malalignment of the knee; absence of hip osteoarthritis; absence of ankle pain; absence of previous fracture on the index knee; absence of previous surgery on the index knee; absence of rheumatoid arthritis; and lack of intra-articular injection in the index knee in the past six months. The patients also needed to have been receiving the usual care for osteoarthritis for at least six months and to be able to understand and agree with the informed consent statement.

### Exclusion criteria

The exclusion criteria for this study were:

- Undergoing surgery during the study period.
- Undergoing intra-articular injection during the study period.
- Developing infection of the index joint during the study period.

One hundred and thirty-eight patients were interviewed, and fifty-eight patients met the inclusion criteria (Figure 1). All the patients in our department receive the same treatment protocol, which we call the usual care for knee osteoarthritis. The usual care consists of patient education through lectures, handouts, audiovisual material and guidance given by orthopedic surgeons, nutritionists, psychologists, occupational therapists, physical therapists, physical educators and social workers. All patients, except those with contraindications, take analgesics (on demand), such as paracetamol and codeine. We do not routinely give non-steroidal anti-inflammatory drug (NSAIDs) to our patients.

All the patients invited to participate in the present study agreed to do so. The study was conducted in an outpatient setting at a tertiary hospital.

One week before start to use the orthotic devices, the patients who met the criteria gave responses on the visual analogue scale for pain (VAS) and to the Western Ontario and McMaster Universities Arthritis Index (WOMAC)^20^ and Lequesne questionnaire.^21^ Anthropometric data was also collected, such as age, gender, race, height, weight and body mass index (BMI). Plain radiographs of the knees were available for all the patients, in anteroposterior view with unilateral weight bearing and in lateral and patellar axial views. Three of us (GCC, TP, RF) examined all the radiographs in order to classify the severity of the osteoarthritis using the Kellgren-Lawrence scheme.^22^ In 18 cases, there was interobserver disagreement. In all of these cases, we took into consideration the classification level given by the majority (two observers). None of the radiographs resulted in total discordance (three different classifications).

The patients were randomly divided into two groups of 29 patients by means of simple randomization. The randomization was performed using a computer-generated program (available from: http://www.randomization.com/) and was done by an investigator who did not have any involvement in the rest of the study. The patients were confidentially allocated to the lateral wedge insole group (Group W) or the neutral insole group (Group N). Although the patients did not know which group they were in, they were not considered to be blind to this, since they could see the shape of the insole.

All the patients used insoles on both feet. Group W patients with unilateral knee osteoarthritis used a lateral wedge insole on the affected limb and a neutral insole on the contralateral limb. Group W patients with bilateral disease used a lateral wedge insole on both limbs. Group N patients used a neutral insole on both limbs.

**Figure 2.**
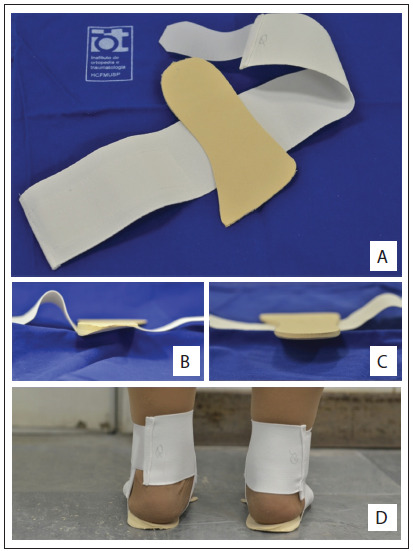
Insoles. (A) Full length ethylene vinyl acetate (EVA) insole attached to an ankle-sprain support; (B) lateral wedge insole (Group W); (C) neutral insole (Group N); (D) patient wearing a lateral wedge insole.

The wedge insoles were made with a full length lateral wedge of 8 mm (equivalent to about eight degrees of inclination) attached to a figure “eight” strap around the ankle (Figure 2). The neutral insoles were exactly the same orthosis, but without a lateral wedge. All the patients were encouraged to use the insoles for 5-10 hours per day.

The VAS, WOMAC and Lequesne questionnaires were again applied during scheduled visits in weeks two, eight and 24. The primary outcomes measured were knee pain and knee function improvement, which were expressed through the results of the questionnaires applied. The secondary outcomes were the presence of adverse effects (such as ankle pain) and any correlation between anthropometric data and clinical outcomes.

The sample size was estimated by calculating n such that it enabled statistical power of 80% and a significance level of 5%. For the primary outcome of VAS, we took the standard deviation (SD) of 1.9 that had been found in a previous study^23^ and stipulated that the difference to be detected should be at least 1.5 points. Using a two-tailed hypothesis test, we found that the number of patients per group should be 25. Taking estimates of 20% for dropouts and exclusions, we calculated that 29 patients per group were needed.

The investigator (MUR) who applied the questionnaires was blinded (unaware of the patient’s group). To determine whether the groups differed with regard to the nominal variables, we used absolute and relative frequencies, and checked for associations using chi-square for gender and the likelihood ratio for race. The Mann-Whitney test was used to compare groups regarding the Kellgren-Lawrence grade. The quantitative characteristics were described in groups through using summary measurements (mean, SD, median, minimum and maximum), and the groups were compared using Student’s t-test. We used a significance level of 5% for all analyses.

## RESULTS

The patients were recruited between June 2011 and July 2011. Twenty-nine patients were randomly assigned to each group, received the intended treatment and were analyzed for functional and pain status using VAS and the WOMAC and Lequesne questionnaires. All the patients were evaluated clinically and started using the lateral wedge insoles between August 2011 and September 2011. The trial ended in March 2012, in week 24 of the follow-up. There were no losses.

There were no differences between the groups in relation to nominal and numerical characteristics (Table 1) or scores (Table 2) at baseline. Scales were also described as groups and times through using summary measurements (Table 3). The relationships among the results were analyzed using Pearson’s correlations regarding numerical characteristics such as age and body mass index (BMI) and using Spearman’s correlation regarding Kellgren-Lawrence grade (Table 4).

**Table 1.**
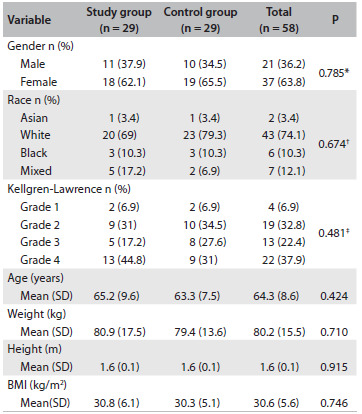
Baseline demographics

P-values from: Student’s t test; ^*^chi-square test; ^†^likelihood ratio; ^‡^Mann-Whitney test

**Table 2.**
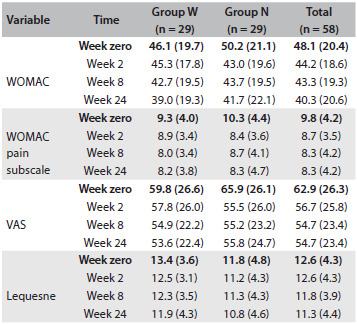
Scores according to groups and times

WOMAC = Western Ontario and McMaster Universities Arthritis Index; VAS = visual analogue scale.

**Table 3.**
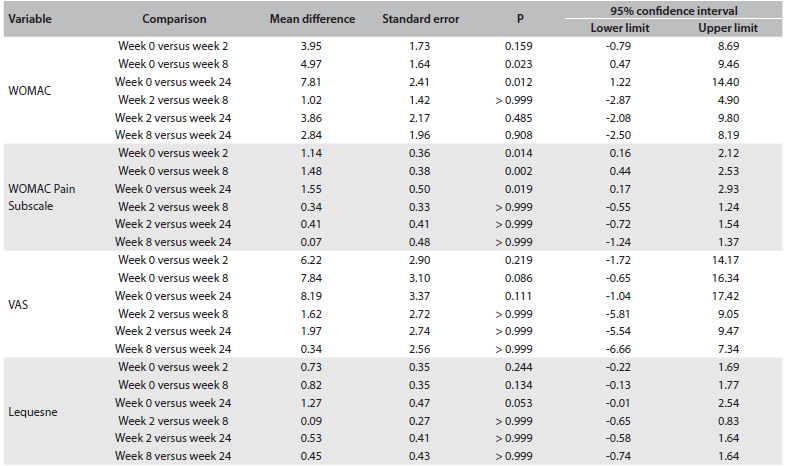
Results from Bonferroni’s multiple comparison

WOMAC = Western Ontario and McMaster Universities Arthritis Index; VAS = visual analogue scale.

**Table 4.**
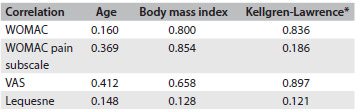
Correlation between scores and particular subgroups (P-values)

Pearson’s correlation; *Spearman’s correlation; WOMAC = Western Ontario and McMaster Universities Arthritis Index; VAS = visual

At weeks 8 and 24, both groups showed lower results for WOMAC, with a difference from baseline (P = 0.023 and P = 0.012 respectively). The mean WOMAC pain subscale score showed statistically significant reductions at all times. in comparison with the baseline, in both groups (P < 0.05). There was no difference between the groups at any time regarding any score (Table 3). There was no correlation between anthropometric data and the clinical outcomes (Table 4). Fifteen percent of all the patients reported ankle discomfort (Table 5). One patient in the W group abandoned the treatment due to ankle pain. There were no differences between the groups regarding adverse effects.

**Table 5.**
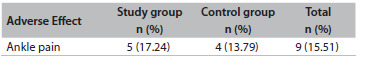
Adverse effects

## DISCUSSION

Biomechanical and clinical studies have shown that lateral wedge insoles can promote a reduction in the load on the medial knee compartment^8^^,^^9^^,^^10^ and symptomatic benefits for patients with medial knee osteoarthritis.^11^^,^^24^ Likewise, medial wedge insoles have shown benefits for patients with lateral compartment knee osteoarthritis.^25^ However, despite recommendations in several guidelines,^26^ neither the present study nor some long-term studies in the literature found any clinical benefit.^17^^,^^18^^,^^23^^,^^27^ To the best of our knowledge, our study is the first clinical trial on lateral wedge insoles in a South American population.

Our study has some limitations. First, we did not limit the use of analgesics or any other non-pharmacological treatment. We believe that use of insoles should not exclude any other type of treatment, and therefore, the patients received their usual care but were asked to keep track of their use of analgesics. No differences were found between the groups in this regard. Second, clinical scores such as WOMAC and Lequesne cannot distinguish one knee from another when the patient has bilateral osteoarthritis. Therefore, patients with bilateral disease had both knees treated and only the knee that was worse (according to the patient) was taken into consideration and classified using the Kellgren-Lawrence grade. Third, our patients had some difficulty in responding to the questionnaires. Our patients were of low educational level and some were illiterate, which may have had a negative impact on the accuracy of the questionnaire responses. We used the WOMAC and Lequesne questionnaires, which have been validated for the Portuguese language.^28^^,^^29^ However, we believe that studies are needed to determine whether low educational level could jeopardize comprehension of these questionnaires.

Variation in daily usage of wedged insoles may also influence the clinical outcome. A non-randomized trial^26^ found that the greatest clinical benefits were obtained from 5-10 hours of daily use, in comparison with less than 5 hours or more than 10 hours. We recommended to our patients that they should use the insoles for 5-10 hours per day, but we are not convinced that this was accomplished, due to extensive use of open footwear such as sandals and flip-flops in Brazilian culture. Our population had trouble using the insoles with open footwear, maybe because of their design (Figure 2).

Toda and Tsukimura^24^ found that a strapped insole, comprising a urethane wedge with a 12-mm elevation that was fixed to an ankle-sprain support, provided clinical benefit. Ours was a full-length insole that was also fixed to an ankle-sprain support, and thus it presented some disadvantages as seen in inserted insoles, such as slipping and changing position. It also presented the disadvantage of the necessity for shoes more than one size larger, to accommodate the thickness of the insole. A combined treatment approach, using elastic subtalar strapping with lateral wedges, reduces the adduction moment more than wedged insoles alone do, particularly in cases of mild and moderate medial osteoarthritis.^10^ This may be because strapping causes valgus angulation of the talus, thereby leading to correction of the femorotibial angle and further reducing the medial joint load.^15^^,^^24^ However, our elderly patients showed difficulty in manipulating the elastic strap, and some were incapable of wearing it without help. The degree of change in the femorotibial angle with the insole with subtalar strapping is affected by the tilt of the lateral wedge.^16^ For constant routine use, wedged insoles with 12-mm elevation and subtalar strapping may be more effective than the 8-mm elevation wedge used in our study.^24^

The present study did not find any correlation between anthropometric data and the clinical outcomes. The cohort studied by Baker et al.^30^ presented high body mass index, with a mean of 33, and a Kellgren-Lawrence score of greater than grade three, with poor results. Conversely, Toda et al.^16^ predominantly recruited females, with low body mass index (mean = 23.5) and Kellgren-Lawrence scores of grade two or three, with substantially better results. Whether these population characteristics are important as confounding variables remains unclear.

At present, there is no evidence to show that lateral wedge insoles are of greater benefit to particular subgroups, such as early or late-stage osteoarthritis or coexisting pathological conditions.^17^ This could be a potential area for future study.

In the present study, both groups showed statistically significant improvement in comparison with the baseline, but with no significant difference within the groups. Therefore, the clinical benefit of this intervention might only have been due to the placebo effect. We also cannot rule out the possibility of type 2 error, meaning that our sample size might not have been large enough to allow adequate statistical analysis.

We do not believe that the lack of improvement in the study group proves that this particular method was totally inefficient. Furthermore, we are certain that, in fact, our results provide an alert regarding several factors that need to be borne in mind when prescribing insoles, such as insole design, insole material, daily usage, cultural factors and subtalar strapping, among others.

## CONCLUSION

We concluded that use of a lateral wedge insole with subtalar strapping improved patients’ symptoms and function but was not superior to use of placebo insoles.
